# Advances in RNA-based therapeutics: current breakthroughs, clinical translation, and future perspectives

**DOI:** 10.3389/fgene.2025.1675209

**Published:** 2025-10-24

**Authors:** Sarbjeet Kaur Makkar

**Affiliations:** ^1^ Division of Hematology and Oncology, Department of Internal Medicine, University of Michigan, Ann Arbor, MI, United States; ^2^ Rogel Cancer Center, University of Michigan, Ann Arbor, MI, United States

**Keywords:** RNA therapeutics, mRNA vaccines, siRNA and antisense oligonucleotides, CRISPR-Cas13, precision medicine

## Abstract

RNA-based therapeutics have revolutionized modern medicine, offering versatile and precise modalities to modulate gene expression for a wide range of diseases including infectious diseases, genetic disorders, and cancer. This review comprehensively examines the evolution and current landscape of RNA therapeutics, encompassing major classes such as mRNA vaccines, small interfering RNAs (siRNAs), antisense oligonucleotides (ASOs), and emerging RNA editing technologies like CRISPR-Cas13. We discuss technological innovations that have overcome historic challenges related to RNA instability, immunogenicity, and delivery, with particular emphasis on lipid nanoparticle formulations and targeted ligand conjugates. Clinical translation milestones, regulatory considerations, and safety profiles are analyzed, highlighting recent approvals and ongoing trials that underscore the therapeutic promise of RNA modalities. Despite these advances, critical challenges including off-target effects, immune activation, manufacturing scalability, and effective delivery to extrahepatic tissues remain to be addressed. The integration of personalized RNA therapeutics, precision RNA editing, and artificial intelligence-driven design and clinical decision support heralds a new era of individualized and adaptive therapies. This synthesis of molecular biology, nanotechnology, and computational innovation not only illustrates the transformative potential of RNA therapeutics but also charts a path toward broad clinical impact and accessible precision medicine. To fully realize this potential, the field must prioritize the development of robust regulatory frameworks and adaptive clinical trial designs to ensure equitable access, while also advancing exploration of emerging modalities such as circular RNAs, self-amplifying RNAs, and RNA-targeting small molecules. Collectively, these innovations are poised to expand the therapeutic landscape beyond current boundaries and accelerate the realization of truly personalized RNA medicine. Looking ahead, progress in RNA therapeutics will depend less on the expansion of new classes alone and more on solving practical challenges in tissue targeting, long-term safety, scalable production, and regulatory adaptation. By comparing established and emerging approaches through these dimensions, this review provides a forward-looking synthesis that identifies where translation is already feasible and where innovation is still required.

## 1 Introduction

The concept of RNA-based therapeutics was pioneered with the development of antisense oligonucleotides in the late 1970s and the discovery of RNA interference in the 1990s, which revealed the ability of RNA to regulate gene expression at the post-transcriptional level (Zamecnik and Stephenson, 1978; Fire et al., 1998). Over the past 2 decades, these early insights have matured into a robust therapeutic platform, with multiple antisense and siRNA drugs achieving regulatory approval and demonstrating clinical benefit in genetic and metabolic diseases (Adams et al., 2018; Ray et al., 2020). The global deployment of mRNA vaccines during the COVID-19 pandemic further validated RNA as a scalable and adaptable modality, capable of rapid response in a public health crisis (Dolgin, 2021). Recent reviews on mRNA nanomedicine further highlight advances in drug delivery systems, molecular stability, and innovative applications that are broadening the scope of RNA therapeutics (Dwivedi et al., 2024; Dwivedi et al., 2025). More recently, advances have extended beyond these established platforms: as shown in Table 1, inclisiran has shown sustained lipid-lowering efficacy in long-term follow-up studies (ORION-4, 2025), eplontersen has demonstrated promise for transthyretin amyloidosis (Benson et al., 2023), and the first CRISPR-based RNA-guided editing therapy, exa-cel, was approved for sickle cell disease (U.S. Food and Drug Administration (FDA), 2023). In parallel, early-phase clinical trials are exploring emerging modalities including self-amplifying RNAs (Bloom et al., 2021), circular RNAs with enhanced stability and translational efficiency (Wesselhoeft et al., 2018; Orna Therapeutics, 2024), and RNA-targeting small molecules. A recent comprehensive review highlights how these innovations are reshaping therapeutic classes, delivery technologies, and clinical translation (Shahid, 2025). Despite this remarkable progress, critical barriers remain—particularly in delivery beyond the liver, long-term safety, large-scale manufacturing, and harmonized regulatory pathways—which continue to define the trajectory of the field.

**TABLE 1:** Selected clinical trials of RNA-based therapeutics.

Therapeutic (Company)	RNA modality	Target/Indication	Trial phase	Status/Key outcome
Patisiran (Onpattro, Alnylam)	siRNA (LNP)	Transthyretin (hATTR amyloidosis)	Phase III (APOLLO)	FDA approved (2018); improved neuropathy scores
Givosiran (Givlaari, Alnylam)	siRNA (GalNAc)	Acute hepatic porphyria	Phase III (ENVISION)	FDA approved (2019); reduced porphyria attacks
Inclisiran (Leqvio, Novartis/Alnylam)	siRNA (GalNAc)	PCSK9/hypercholesterolemia	Phase III (ORION trials)	FDA approved (2021); LDL-C reduction sustained >18 months
Eplontersen (Ionis/AstraZeneca)	ASO	Transthyretin amyloidosis	Phase III (NEURO-TTRansform)	Positive interim results (2024); regulatory submission expected
Nusinersen (Spinraza, Biogen/Ionis)	ASO (Splice-switching)	SMN2 splicing/spinal muscular atrophy	Phase III (ENDEAR, CHERISH)	FDA approved (2016); improved motormilestones and survival
Casgevy (Exa-cel, Vertex/CRISPR Tx)	CRISPR-Cas9 gene editing	β-thalassemia, sickle cell disease	Phase III	FDA approved (2023); functional cure in majority of patients
mRNA-4157 (Moderna/Merck)	mRNA cancer vaccine	Personalized neoantigens (melanoma)	Phase IIb	Reported significant recurrence-free survival benefit (2023); Phase III planned
mRNA-1345 (Moderna)	mRNA vaccine	RSV (older adults)	Phase III	Positive results; FDA priority review (2024)
Self-amplifying RNA (saRNA, Arcturus/Imperial College)	saRNA vaccine	COVID-19 and influenza	Phase II/III	Demonstrated durable antibody response; multi-pathogen vaccines in progress (2024–2025)
Circular RNA cancer vaccine (preclinical → Phase I, Orna Therapeutics)	circRNA	Oncology applications	Phase I (2024)	First-in-human trial initiated; early safety data awaited

Subsequent research expanded upon these foundational studies by exploring the mechanisms and potential therapeutic applications of various RNA modalities, including antisense oligonucleotides (ASOs), small interfering RNA (siRNA), microRNA (miRNA), and messenger RNA (mRNA) (Crooke, 2017; Elbashir et al., 2001; Ambros, 2004). Early antisense technologies were developed to selectively inhibit mRNA translation, providing a promising therapeutic avenue for diseases driven by aberrant protein production (Crooke, 2017). In parallel, siRNA emerged as a powerful tool capable of inducing robust and highly specific gene silencing, subsequently validated by numerous preclinical studies demonstrating its potential for treating genetic and viral diseases (Elbashir et al., 2001). Despite these encouraging developments, significant technical hurdles, including RNA instability, rapid enzymatic degradation, immune recognition, and inefficient cellular delivery, initially hindered clinical progress (Whitehead et al., 2009; Kim and Rossi, 2008). To address these challenges, extensive research efforts were directed toward chemical modifications of RNA molecules, formulation optimization, and the development of efficient delivery vehicles such as lipid nanoparticles (LNPs), polymeric nanoparticles, and viral vectors (Cullis and Hope, 2017; Hou et al., 2021).

This transformation was dramatically underscored by the swift and highly successful development of mRNA-based vaccines during the COVID-19 pandemic, such as those produced by Pfizer-BioNTech and Moderna, which demonstrated remarkable efficacy and safety in combating SARS-CoV-2 infection (Baden et al., 2021; Polack et al., 2020). These landmark achievements validated the practical applicability of mRNA therapeutics, positioning RNA-based approaches at the forefront of translational medicine.Today, RNA therapies encompass diverse modalities, from classical approaches like ASOs and siRNAs to emerging innovations such as RNA editing and CRISPR-based RNA targeting (Abudayyeh et al., 2017; Adams et al., 2018; Mercuri et al., 2018). Clinical milestones, such as the FDA approval of the siRNA therapeutic Patisiran for transthyretin amyloidosis, further underscore the substantial clinical potential and transformative capabilities of RNA interference-based therapeutics (Adams et al., 2018).Furthermore, recent advancements in RNA chemistry, including nucleotide modifications and sequence optimization, have significantly improved stability, reduced immunogenicity, and enhanced translational efficiency, facilitating broader clinical adoption (Jackson et al., 2020). Despite these advances, critical challenges persist, notably immunogenic responses, off-target effects, and complexities in scaling up manufacturing processes (Kis et al., 2021; Crommelin et al., 2021).

This comprehensive review critically evaluates the current state of RNA-based therapeutics, detailing significant advancements across various RNA modalities, analyzing the technological breakthroughs enabling their clinical translation, and discussing ongoing regulatory considerations. Additionally, it highlights unresolved challenges and provides a forward-looking perspective on future opportunities, particularly emphasizing personalized medicine, novel RNA editing technologies, and the integration of artificial intelligence (AI) in advancing RNA therapeutic strategies (Angermueller et al., 2016; Nishikura, 2010; Sahin and Türeci, 2018). The detailed analysis provided herein aims to guide researchers and clinicians alike, promoting continued innovation and collaboration essential for fully realizing the vast therapeutic potential of RNA-based treatments.

## 2 Major classes of RNA therapeutics

### 2.1 mRNA vaccines

Messenger RNA (mRNA) vaccines represent a transformative approach in vaccinology, exemplified by their rapid development and deployment during the COVID-19 pandemic. Early research on mRNA vaccines dates back to the 1990s, but progress was initially hindered by instability issues, immunogenicity, and inefficient delivery systems (Wolff et al., 1990; Hoerr et al., 2000) Figure 1. However, breakthroughs in nucleotide modifications, specifically the incorporation of modified nucleosides such as pseudouridine, significantly improved the stability and reduced the immune activation of mRNA, facilitating effective clinical application (Karikó et al., 2005a; Karikó and Weissman, 2007). The mRNA vaccines developed by Pfizer-BioNTech and Moderna demonstrated unprecedented efficacy and rapid production timelines, marking a new era in vaccine technology (Polack et al., 2020; Baden et al., 2021). These vaccines function by delivering synthetically produced mRNA encoding the SARS-CoV-2 spike protein, inducing host cells to transiently produce viral antigens and stimulate robust immune responses without introducing an infectious agent (Jackson et al., 2020). Beyond infectious diseases, mRNA vaccines have also shown promising potential in oncology as personalized cancer vaccines, wherein patient-specific tumor antigens encoded by mRNA elicit tailored immune responses against cancer cells (Sahin and Türeci, 2018).

**FIGURE 1:**
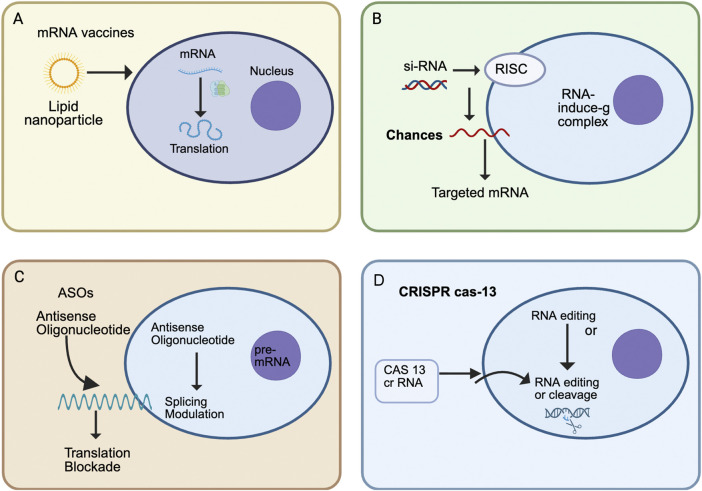
Overview of major RNA therapeutic modalities. **(A)** mRNA vaccines use lipid nanoparticles (LNPs) to deliver synthetic mRNA into cells, where ribosomes translate it into antigenic proteins for immune priming. **(B)** Small interfering RNAs (siRNAs) associate with the RNA-induced silencing complex (RISC) to guide sequence-specific degradation of target mRNAs, thereby reducing disease-causing protein expression. **(C)** Antisense oligonucleotides (ASOs) bind to complementary mRNA or pre-mRNA sequences to modulate splicing or block translation, offering therapeutic benefits in genetic and neuromuscular disorders. **(D)** CRISPR-Cas13 systems employ programmable CRISPR RNAs to edit or cleave target RNAs directly, enabling transient and reversible gene modulation without altering the DNA genome.

### 2.2 siRNA therapeutics

Small interfering RNAs (siRNAs) exploit the endogenous RNA interference (RNAi) pathway discovered initially in plants and later elucidated in animals, highlighting a conserved mechanism across species for gene silencing (Fire et al., 1998; Elbashir et al., 2001). Clinical validation of siRNA therapeutics was significantly advanced by the approval of Patisiran, which targets transthyretin mRNA to treat hereditary transthyretin-mediated amyloidosis (Adams et al., 2018). Similarly, Givosiran, an approved siRNA therapy for acute hepatic porphyria, underscores the versatility and therapeutic potential of siRNAs in treating metabolic disorders by precisely silencing pathogenic genes (Balwani et al., 2020). Despite their clinical successes, siRNA therapeutics continue to face challenges such as delivery efficiency, immune activation, and specificity, which ongoing research aims to overcome through advanced chemical modifications and novel delivery platforms such as lipid nanoparticles (LNPs) (Setten et al., 2019; Kulkarni et al., 2018).

### 2.3 Antisense oligonucleotides (ASOs)

Antisense oligonucleotides (ASOs) are short synthetic RNA or DNA molecules designed to hybridize specifically to complementary mRNA sequences, modulating their processing or translation. Initial discoveries in antisense technologies occurred in the late 1970s and early 1980s, paving the way for modern therapeutics (Zamecnik and Stephenson, 1978). Spinraza, an ASO approved for spinal muscular atrophy, alters the splicing of survival motor neuron 2 (SMN2) pre-mRNA to increase the production of functional SMN protein, significantly improving patient outcomes (Mercuri et al., 2018). Another no example, Eteplirsen, approved for Duchenne muscular dystrophy, employs exon-skipping technology to restore the dystrophin reading frame and produce functional dystrophin protein (Mendell et al., 2016). Continuous improvements in ASO design, stability, and delivery are critical to further expand their therapeutic applications across diverse genetic disorders (Crooke, 2017).

### 2.4 Emerging RNA modalities (CRISPR-Cas RNA targeting)

Emerging RNA therapeutic modalities, notably CRISPR-based RNA targeting, have opened novel avenues for precision medicine. The discovery of the CRISPR-Cas system as a bacterial adaptive immune mechanism has revolutionized genome editing technologies (Jinek et al., 2012). The CRISPR-Cas13 system, in particular, allows specific RNA targeting and cleavage without affecting DNA, offering transient and reversible gene expression modulation (Abudayyeh et al., 2017). This RNA-editing technology has demonstrated potential in targeting viral RNAs, correcting disease-associated transcripts, and modulating gene expression with high specificity and minimal off-target effects (Gootenberg et al., 2017). Ongoing research aims to optimize CRISPR-Cas13 efficacy, specificity, and delivery methods, further expanding its potential for diverse therapeutic applications in viral infections, genetic disorders, and beyond (Pickar-Oliver and Gersbach, 2019).

## 3 Technological innovations and delivery mechanisms

**FIGURE 2:**
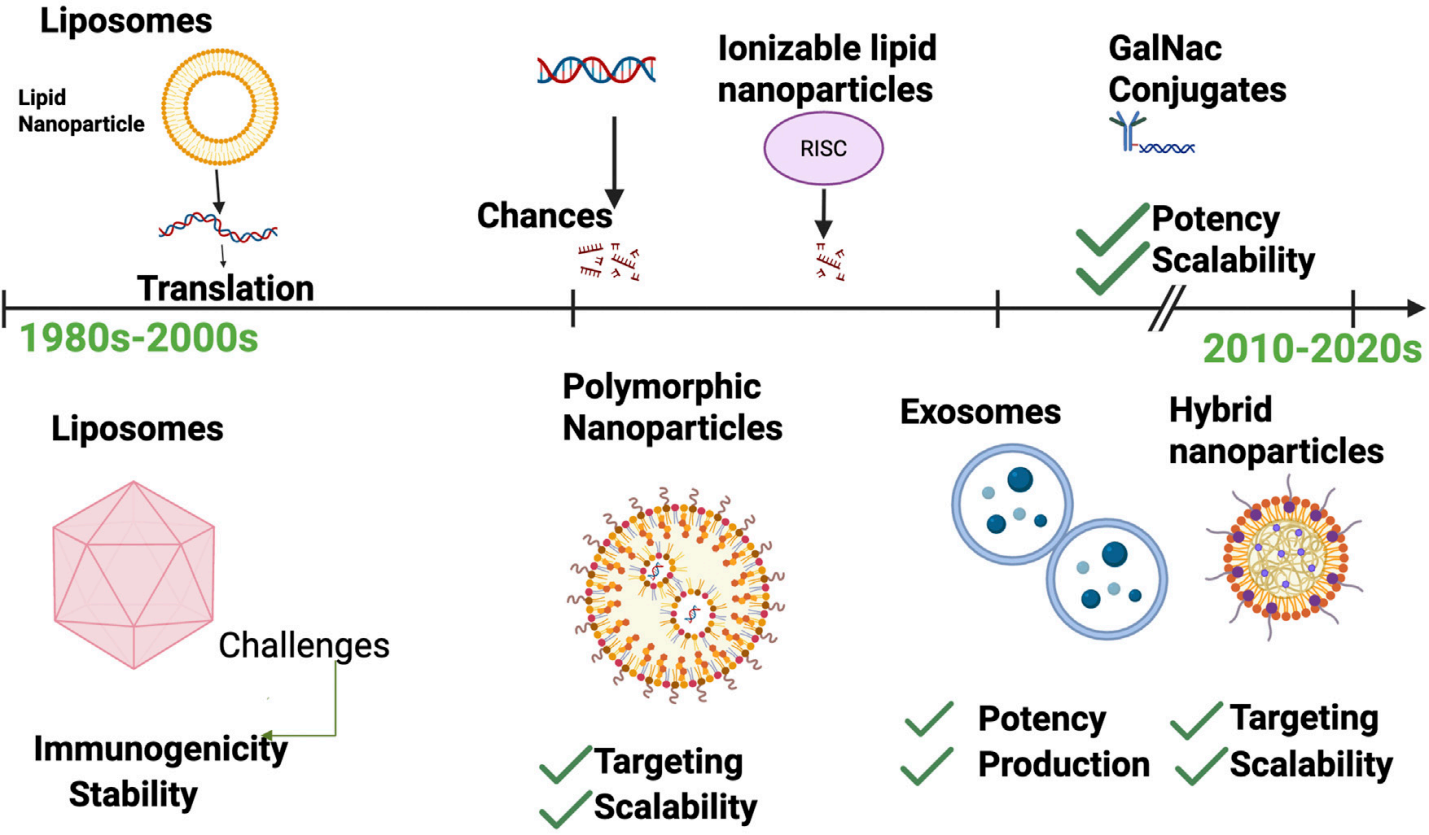
Timeline of RNA Delivery Technologies. Chronological progression from early liposome-based delivery in the 1980s to advanced hybrid systems in the 2020s. Key milestones include (i) first liposome-mediated gene transfer (1987), (ii) development of ionizable lipid nanoparticles enabling mRNA vaccines and siRNA drugs, (iii) GalNAc conjugates achieving targeted hepatocyte delivery, and (iv) exosome-based delivery currently in preclinical development. Advantages and limitations of each platform are noted to highlight trade-offs between efficiency, safety, and scalability.

The therapeutic potential of RNA molecules has long been recognized, but their clinical application was historically limited by challenges related to instability, immunogenicity, and inefficient delivery to target cells. Over the last few decades, significant technological innovations have transformed RNA delivery, enabling effective protection, targeting, and controlled release of RNA therapeutics Figure 2.

### 3.1 Lipid nanoparticles (LNPs)

Lipid nanoparticles have become the most widely utilized platform for RNA delivery, particularly highlighted by their success in COVID-19 mRNA vaccines (Hou et al., 2021). The concept of lipid-based delivery dates back to studies in the 1980s exploring liposome-mediated gene transfer (Felgner et al., 1987). Early RNA delivery attempts suffered from rapid degradation by extracellular RNases and poor cellular uptake (Whitehead et al., 2009). The breakthrough came with the design of ionizable lipids that are neutral at physiological pH but become positively charged in acidic environments such as endosomes, facilitating endosomal escape and cytoplasmic RNA release (Cullis and Hope, 2017). Ionizable lipid formulations reduce toxicity and immunogenicity compared to permanently cationic lipids (Heyes et al., 2005). In addition to ionizable lipids, LNPs contain helper lipids, cholesterol, and polyethylene glycol (PEG)-lipids to optimize stability and circulation time (Sahay et al., 2020).

Clinically, LNPs enable high RNA encapsulation efficiency and favorable pharmacokinetics, leading to robust protein expression in target tissues (Kulkarni et al., 2018). The Pfizer-BioNTech and Moderna vaccines used LNPs with optimized ionizable lipids to safely deliver SARS-CoV-2 spike protein-encoding mRNA, illustrating the real-world success of this technology (Polack et al., 2020; Baden et al., 2021). Beyond vaccines, LNPs are employed for siRNA drugs such as Onpattro (Patisiran) for hereditary transthyretin amyloidosis, further validating their versatility (Adams et al., 2018).

However, challenges remain, including potential PEG immunogenicity leading to accelerated blood clearance upon repeated dosing and limitations in tissue targeting beyond the liver (Irvine et al., 2020). Recent research focuses on novel lipid chemistries, biodegradable ionizable lipids, and incorporation of targeting ligands to improve delivery specificity and reduce adverse effects (Lonez et al., 2014; Cheng et al., 2020).

### 3.2 Viral vectors

Viral vectors, including adenoviruses, adeno-associated viruses (AAV), and lentiviruses, offer efficient gene delivery by exploiting natural viral infection mechanisms (Naldini, 2015). While predominantly used for DNA delivery, these vectors have also been adapted for RNA delivery in some contexts. Their advantages include high transduction efficiency and potential for long-term expression, crucial for certain therapeutic indications (Mingozzi and High, 2013). However, viral vectors face significant limitations: immunogenicity triggering neutralizing antibodies and inflammation, limited packaging capacity (particularly for AAVs), complex manufacturing, and safety concerns related to insertional mutagenesis (Thomas et al., 2003; Ginn et al., 2018). The immune response against viral vectors often restricts repeat dosing, posing challenges for chronic therapies (Shirley et al., 2020). Engineering less immunogenic viral capsids, use of serotype switching, and transient immunosuppression strategies are being explored to mitigate these challenges (Kotterman and Schaffer, 2014).

### 3.3 Polymeric nanoparticles and other emerging delivery systems

Polymeric nanoparticles represent one of the most versatile classes of RNA delivery vehicles due to their tunable chemistry, biodegradability, and potential for controlled release. Commonly studied polymers include poly (lactic-co-glycolic acid) (PLGA), polyethylenimine (PEI), poly (β-amino esters) (PBAEs), and dendrimers, each offering distinct physicochemical properties that can be tailored for nucleic acid encapsulation (Danhier et al., 2012; Verbaan et al., 2005). PLGA nanoparticles, already FDA-approved for other drug formulations, provide favorable safety profiles and sustained release kinetics, but often require surface modifications (e.g., PEGylation or ligand conjugation) to enhance cellular uptake and protect RNA cargo. In contrast, cationic polymers like PEI demonstrate high transfection efficiency through strong electrostatic interactions with RNA, yet are limited by dose-dependent cytotoxicity and variable *in vivo* performance (Mintzer and Simanek, 2009). To overcome these limitations, biodegradable or low-molecular-weight PEI derivatives and PBAEs have been engineered to balance transfection efficiency with reduced toxicity (Sunshine et al., 2012).

Emerging approaches also leverage stimuli-responsive polymers, which release RNA cargo in response to pH, redox state, or enzymatic activity within target tissues, improving precision and minimizing systemic side effects (Rosenblum et al., 2020). Hybrid systems that integrate polymeric and lipid components are gaining attention for combining the stability and functionalization capacity of polymers with the high encapsulation efficiency of lipids (Wang et al., 2018).

Beyond synthetic polymers, biological carriers such as exosomes and extracellular vesicles (EVs) offer natural RNA transport mechanisms, with intrinsic tissue tropism and low immunogenicity (El Andaloussi et al., 2013). Preclinical studies demonstrate successful delivery of mRNA and siRNA using engineered exosomes; however, reproducible large-scale manufacturing and standardized loading methods remain major hurdles (Lener et al., 2015; Armstrong and Stevens, 2018). Although still in early translational stages compared to lipid nanoparticles, polymeric and exosome-based delivery systems hold unique promise for extrahepatic targeting, repeat dosing, and crossing biological barriers such as the blood–brain barrier. Continued progress will likely depend on integrating polymer chemistry, bioengineering, and scalable manufacturing to achieve clinically viable platforms.

### 3.4 Chemical modifications and targeting strategies

Chemical modification of RNA molecules has been pivotal in improving stability and reducing innate immune activation (Karikó et al., 2005b). Incorporation of modified nucleotides such as pseudouridine and 5-methylcytidine reduces recognition by Toll-like receptors and RIG-I, enhancing translational efficiency and reducing inflammation (Anderson et al., 2010; Svitkin et al., 2017). Concurrently, conjugation of RNA molecules or their delivery vehicles with targeting ligands has facilitated cell- and tissue-specific delivery, thereby increasing therapeutic indices and reducing systemic toxicity. Ligands such as N-acetylgalactosamine (GalNAc), aptamers, and antibodies enable selective binding to receptors on target cells, promoting efficient uptake primarily through receptor-mediated endocytosis (Nair et al., 2014; Whitehead et al., 2014). Notably, GalNAc conjugation has been extensively utilized for hepatocyte targeting via the asialoglycoprotein receptor (ASGPR), a strategy that underpins several clinically approved siRNA therapeutics for liver diseases.

#### 3.4.1 Chemical modifications of RNA

Pioneering studies by Karikó and Weissman in the mid-2000s demonstrated that incorporating modified nucleosides, such as pseudouridine (Ψ) and 5-methylcytidine (m5C), into synthetic RNA molecules significantly reduces activation of immune sensors, enabling more efficient translation and reduced toxicity (Karikó et al., 2005a; Karikó and Weissman, 2007). These modifications help evade immune recognition by Toll-like receptors, specifically TLR3, TLR7, and TLR8, which are known to detect foreign RNA species (Hornung et al., 2006). Moreover, modified nucleotides improve the chemical stability of RNA by enhancing resistance to nucleases, extending circulation time and allowing for effective dosing (Nance and Meier, 2021).

Further chemical optimization includes backbone modifications such as phosphorothioate linkages, which confer resistance to exonucleases and endonucleases, and sugar modifications like 2′-O-methyl and 2′-fluoro modifications that improve stability and binding affinity to target RNA (Crooke, 2017; Yu et al., 2004). These modifications are prevalent in clinically approved antisense oligonucleotides (ASOs) and siRNA drugs, balancing increased stability with maintained biological activity.

#### 3.4.2 Targeting strategies

Targeting RNA therapeutics to specific cell types or tissues enhances therapeutic efficacy while minimizing off-target effects and systemic toxicity. One of the most successful targeting approaches involves conjugating RNA molecules or delivery vehicles with ligands that bind selectively to receptors expressed on target cells. A prominent example is the conjugation of N-acetylgalactosamine (GalNAc) to siRNA molecules, which enables highly efficient and specific delivery to hepatocytes through binding to the asialoglycoprotein receptor (ASGPR) (Nair et al., 2014). GalNAc-siRNA conjugates have demonstrated robust gene silencing in liver cells with minimal systemic exposure, leading to several FDA-approved therapies for liver-associated diseases (Hua et al., 2017).

Other targeting ligands include antibodies, aptamers, peptides, and small molecules that can be linked to nanoparticles or directly to RNA molecules. Aptamers, short single-stranded nucleic acids that fold into specific three-dimensional structures, can bind to a wide range of cellular receptors, offering versatile and highly specific targeting (Zhou and Rossi, 2017). Peptide ligands facilitate receptor-mediated endocytosis and have been utilized to enhance delivery to tumors, immune cells, and the central nervous system (Xue et al., 2019). Additionally, the surface functionalization of lipid nanoparticles and polymeric carriers with targeting moieties further refines tissue-specific delivery. For example, attaching antibodies or receptor-targeting peptides on the surface of LNPs enables selective uptake by cancer cells or immune cell subsets (Siegwart et al., 2011; Pardi et al., 2018).

#### 3.4.3 Controlled release and stimuli-responsive systems

Advanced delivery platforms incorporate stimuli-responsive features that enable controlled release of RNA cargo in response to environmental cues such as pH, redox conditions, or enzymatic activity specific to the target tissue or cellular compartment (Rosenblum et al., 2020). For instance, pH-sensitive lipids or polymers can destabilize endosomal membranes in the acidic endosomal environment, facilitating RNA release into the cytoplasm (Yin et al., 2014). Redox-sensitive linkages respond to the high glutathione concentration inside cells to trigger cargo release (Zhu et al., 2018). These smart delivery systems enhance the precision and efficiency of RNA therapeutics.

When evaluated side by side, each delivery platform reveals distinct translational trade-offs. LNPs are highly scalable and sequence-agnostic but remain primarily hepatocyte-directed, with only limited readiness for extrahepatic applications (Table 2). GalNAc conjugates achieve excellent precision and repeat-dosing feasibility but are restricted to the liver. AAV vectors provide durable expression and engineered serotypes allow tissue targeting, yet their high cost and limited re-dosing capacity constrain broader use. Polymeric nanoparticles are chemically versatile and biodegradable but show variable efficiency and safety *in vivo*. Exosomes offer natural biocompatibility and barrier-crossing potential, but large-scale production and quality control remain unresolved. These contrasts are summarized in Table 2, which benchmarks delivery systems against translational dimensions such as extrahepatic readiness and re-dosing feasibility.

**TABLE 2:** Comparative overview of RNA delivery platforms highlighting their advantages, limitations, clinical validation, translational hurdles, and feasibility for re-dosing and extrahepatic applications.

Delivery platform	Advantages	Limitations	Clinical success examples	Translational challenges	Readiness beyond liver	Re-dosing feasibility
Lipid nanoparticles (LNPs)	High encapsulation efficiency; scalable manufacturing; sequence-agnostic	Liver-biased biodistribution; innate immune activation; PEG-related reactions in some patients	mRNA COVID-19 vaccines; patisiran (siRNA)	Endosomal escape; improved extrahepatic targeting needed	Low–Moderate	Yes (innate activation manageable)
GalNAc conjugates	Precise hepatocyte targeting; convenient SC dosing	Restricted to hepatocytes; limited ligand diversity for other tissues	Inclisiran, givosiran, lumasiran	Development of ligands for non-hepatic tissues	Low	Yes (well tolerated, chronic dosing established)
AAV vectors	Durable expression; broad tissue tropism via serotypes	Pre-existing immunity; limited re-dosing; high production cost	Several approved gene therapies (DNA delivery)	Immunogenicity; large-scale GMP production	Moderate (tissue-dependent)	Limited (neutralizing antibodies constrain repeat use)
Polymeric nanoparticles	Tunable chemistry; potential for biodegradable carriers	Variable efficiency; safety and batch consistency concerns	Early clinical exploration	Standardization; reproducibility	Low	Yes (dose-dependent)
Exosomes/EVs	Biomimetic; can cross biological barriers	Heterogeneity; scalability and QC challenges	Preclinical and early-phase studies	Large-scale isolation; potency assays	Low	Unknown (still under investigation)

## 4 Clinical translation and regulatory milestones

The clinical translation of RNA therapeutics has undergone a remarkable transformation from early experimental concepts to approved therapies impacting diverse medical fields. This progress rests on decades of foundational research into RNA biology, nucleic acid chemistry, and delivery systems, combined with evolving regulatory frameworks that balance innovation and patient safety.

### 4.1 Foundational studies and early trials

The concept of RNA interference (RNAi) was first elucidated in *Caenorhabditis elegans* by Fire et al., in 1998, demonstrating potent, sequence-specific gene silencing via double-stranded RNA (Fire et al., 1998). This discovery catalyzed the exploration of synthetic small interfering RNAs (siRNAs) as therapeutics. Early delivery hurdles were addressed by chemical stabilization strategies (e.g., phosphorothioate backbones) and nanoparticle encapsulation to protect siRNAs from nuclease degradation (Whitehead et al., 2009). The first clinical trial of an siRNA drug, ALN-VSP, targeted liver cancer using LNP delivery, laying groundwork for RNAi therapeutics (Zimmermann et al., 2006). However, systemic administration raised challenges related to off-target effects and immune stimulation (Judge et al., 2006). The subsequent FDA approval of Patisiran in 2018 validated these early efforts by demonstrating safe and effective delivery of siRNA via LNPs to hepatocytes, with meaningful clinical improvement in hereditary transthyretin amyloidosis (Adams et al., 2018). Antisense oligonucleotides (ASOs), first described in the late 1970s by Zamecnik and Stephenson, also faced initial challenges in stability and delivery (Zamecnik and Stephenson, 1978). Through extensive chemical modification (2′-O-methyl, phosphorothioate) and optimization, ASOs like Nusinersen have become standard of care for spinal muscular atrophy, highlighting their clinical viability (Mercuri et al., 2018; Crooke, 2017).

### 4.2 The mRNA vaccine breakthrough and accelerated clinical timelines

The advent of mRNA vaccines represents a landmark in RNA therapeutic development, accelerated dramatically by the urgency of the COVID-19 pandemic. While mRNA technology had been under investigation for decades, its clinical translation was limited by RNA instability and immunogenicity (Wolff et al., 1990; Karikó et al., 2005b). Key innovations—such as nucleoside modifications that diminish innate immune recognition and lipid nanoparticle (LNP) delivery systems that enhance cellular uptake and protect mRNA—catalyzed breakthroughs (Cullis and Hope, 2017; Anderson et al., 2010). The Pfizer-BioNTech (BNT162b2) and Moderna (mRNA-1273) vaccines exemplify these advancements. Both vaccines utilize nucleoside-modified mRNA encoding the SARS-CoV-2 spike protein, delivered via optimized LNPs. Clinical trials demonstrated approximately 95% efficacy in preventing symptomatic COVID-19 with acceptable safety profiles (Polack et al., 2020; Baden et al., 2021). These trials were completed within months, a pace previously unparalleled in vaccine development.

Regulatory agencies leveraged Emergency Use Authorizations (EUAs) to expedite access, balancing rapid review with rigorous safety assessments (U.S. Food and Drug Administration (FDA), 2020). Post-marketing surveillance has provided real-world validation of vaccine safety and effectiveness, despite rare adverse events such as myocarditis, primarily in young males (Mevorach et al., 2021). This success story validates mRNA platforms for rapid response to emergent pathogens and establishes a blueprint for future vaccine development, including for cancer and other infectious diseases (Sahin and Türeci, 2018; Pardi et al., 2018).

### 4.3 Regulatory frameworks: challenges and adaptations

RNA therapeutics challenge traditional regulatory paradigms, requiring adaptation to their unique manufacturing and safety profiles. Regulatory guidance documents, such as those from the FDA and EMA, emphasize stringent characterization of RNA integrity, purity, and the physicochemical properties of delivery systems, particularly lipid nanoparticles (FDA, 2018; European Medicines Agency (EMA), 2025).

Quality control for RNA therapeutics includes assessment of RNA sequence fidelity, capped structures, and chemical modifications, which impact stability and immunogenicity (Kauffman et al., 2016). Analytical methods such as next-generation sequencing (NGS), capillary electrophoresis, and mass spectrometry are routinely employed for lot release and stability studies. Nonclinical safety assessments focus on immunogenicity, off-target effects, and biodistribution, leveraging *in vitro* and *in vivo* models to predict human responses (Setten et al., 2019). Regulatory agencies increasingly encourage incorporation of pharmacogenomics and biomarker studies in clinical trials to identify responders and monitor adverse events.The rapid clinical development of COVID-19 mRNA vaccines underscored the utility of flexible regulatory pathways, including adaptive trial designs, rolling reviews, and conditional approvals (U.S. Food and Drug Administration (FDA), 2020). However, these accelerated frameworks also highlight the need for robust pharmacovigilance systems to monitor long-term safety and efficacy post-approval.

### 4.4 Safety challenges and risk mitigation

The inherent immunostimulatory properties of RNA pose significant safety considerations. Unmodified RNA activates innate immune receptors including Toll-like receptors (TLR3, TLR7, TLR8) and RIG-I-like receptors, potentially inducing inflammatory cytokines and systemic reactions (Hornung et al., 2006; Karikó et al., 2005a). Chemical modifications such as pseudouridine and 5-methylcytidine reduce these responses by altering recognition patterns (Anderson et al., 2010; Svitkin et al., 2017). Delivery systems can also provoke immune responses. PEGylated lipids, used to stabilize LNPs, have been associated with rare hypersensitivity reactions and accelerated blood clearance upon repeat dosing, necessitating ongoing optimization (Irvine et al., 2020).

Off-target gene silencing, a concern primarily for siRNA and ASO therapies, can result in unintended downregulation of genes leading to toxicity. Advanced bioinformatics, transcriptome-wide binding studies, and chemical modifications reduce this risk (Jackson et al., 2016; Setten et al., 2019). Additionally, rigorous clinical monitoring for organ toxicities, particularly hepatic and renal, is standard practice. Emerging gene-editing RNA therapies, such as CRISPR-Cas9 systems, require detailed off-target analysis to preempt genome instability or mutagenesis (Gillmore et al., 2021). Safety monitoring protocols incorporate next-generation sequencing to detect unintended edits, combined with long-term follow-up for genotoxicity.

A major policy gap exists in the regulation of *n-of-1* or ultra-personalized RNA therapeutics. The rapid development of Milasen, an antisense oligonucleotide created for a single patient with Batten disease (Kim et al., 2019), underscores both the feasibility and the regulatory vacuum in such scenarios. Current FDA and EMA frameworks do not yet provide clear, standardized pathways for compassionate use or accelerated approval of bespoke RNA therapies, raising ethical questions around equity, cost, and long-term monitoring. Proposed frameworks include adaptive trial designs, patient registries for real-world data capture, and policy harmonization across jurisdictions to ensure that individualized therapies can be developed safely while remaining accessible.

### 4.5 Future perspectives and emerging RNA modalities

The landscape of RNA therapeutics continues to expand with innovations such as self-amplifying RNA (saRNA), circular RNA (circRNA), and novel gene-editing approaches. saRNA leverages replicon technology to amplify intracellular RNA, allowing lower doses and prolonged protein expression, currently in early clinical development (Alameh et al., 2021). circRNAs offer enhanced stability due to their covalently closed loop structures, representing promising candidates for sustained protein production (Wesselhoeft et al., 2018).

CRISPR-based therapies are progressing rapidly, with *in vivo* genome editing trials for genetic disorders such as transthyretin amyloidosis demonstrating safety and efficacy (Gillmore et al., 2021). Delivery challenges remain significant for gene editors, driving research into improved lipid and viral vector platforms. Artificial intelligence and machine learning are increasingly integrated into RNA drug discovery and safety profiling, accelerating candidate optimization and enabling personalized therapeutics. Regulatory frameworks are anticipated to evolve in tandem with these advances, ensuring patient safety while promoting innovation.

## 5 Current challenges and limitations

Despite the rapid evolution and clinical successes of RNA therapeutics, several intrinsic and extrinsic challenges remain that limit their universal application. Immunogenicity, off-target effects, and manufacturing scale-up complexities pose significant hurdles in translating RNA-based modalities into widely accessible treatments (Table 3). Addressing these limitations requires concerted efforts in molecular design, delivery technologies, regulatory oversight, and manufacturing innovation.

**TABLE 3:** Summary of key translational barriers in RNA therapeutics, outlining biological challenges, current mitigation strategies, and future directions across delivery modalities.

Challenge	Underlying issues	Current approaches	Limitations	Future directions	Modalities most impacted	Evidence maturity
Immunogenicity	Activation of innate sensors (TLRs, RIG-I, PKR); PEG antibodies	Nucleoside modifications; lipid optimization	Residual immune activation with repeat dosing	Biodegradable lipids; tolerance-inducing carriers	mRNA (LNPs), saRNA, repeat-dosed ASOs	Clinical
Off-target effects	Sequence similarity; unintended transcript binding	In-silico design; stereopure chemistries	Off-target silencing remains possible	Transcriptome-wide screening; AI-guided design	siRNA, ASOs, CRISPR-based editors	Clinical/Early clinical
Manufacturing scalability	Cost, GMP capacity, QC	Established for mRNA (LNP); improving for AAV	Viral vectors remain costly; exosomes lack standards	Continuous GMP; harmonized global pipelines	AAV > exosomes/polymers > LNPs	Clinical/Early clinical
Delivery beyond liver	Hepatocyte tropism bias of GalNAc/LNP	Exploratory ligands, exosomes, polymeric carriers	Inefficient targeting in CNS, lung, tumors	Hybrid carriers; ligand libraries	All non-hepatic applications	Preclinical/Early clinical
Long-term safety	Chronic dosing; immune memory; durability of editing	Long-term monitoring; chemistry refinement	Sparse long-horizon safety data	Patient registries; adaptive clinical trials	mRNA vaccines (boosting), chronic siRNA/ASO, CRISPR editors	Early clinical

### 5.1 Immunogenicity: molecular and delivery-induced innate immune activation

Immunogenicity is among the foremost challenges confronting RNA therapeutics. Exogenous RNA molecules are recognized by innate immune receptors, including endosomal Toll-like receptors (TLR3, TLR7, TLR8) and cytosolic sensors such as RIG-I and MDA5, which detect pathogen-associated molecular patterns and trigger pro-inflammatory cascades (Hornung et al., 2006; Kawai and Akira, 2008). The resulting activation of type I interferon pathways can lead to systemic inflammation, cytokine release syndrome, and attenuation of therapeutic efficacy due to rapid RNA clearance (Karikó et al., 2005b; Robbins et al., 2020).

Chemical modification of RNA nucleosides, notably the incorporation of pseudouridine, 5-methylcytidine, and N1-methyl-pseudouridine, has proven effective in mitigating immunogenicity by reducing recognition by these pattern recognition receptors, thus enhancing translational capacity and reducing inflammatory side effects (Karikó et al., 2005a; Anderson et al., 2010; Svitkin et al., 2017). However, even chemically modified RNAs are not entirely inert, especially with repeated administrations, where immune memory can exacerbate responses (Kaczmarek et al., 2017). Additionally, delivery vehicles contribute to immunogenicity. Polyethylene glycol (PEG) moieties used in lipid nanoparticle formulations prolong circulation but can induce anti-PEG antibodies, causing accelerated blood clearance (ABC phenomenon) and hypersensitivity reactions (Irvine et al., 2020). Biodegradable lipid components and alternative stealth strategies are under active investigation to circumvent these issues (Kulkarni et al., 2018).

While chemical modifications such as pseudouridine incorporation reduce recognition by innate immune receptors, they do not fully eliminate immune activation, particularly with repeat dosing (Karikó et al., 2005b; Kaczmarek et al., 2017). This suggests that future progress requires not only molecular modifications but also systemic strategies for immune tolerance induction, including biodegradable lipid carriers and transient immunosuppression protocols. Without such measures, the durability of chronic RNA therapies will remain limited.

### 5.2 Off-target effects: molecular specificity and transcriptomic complexity

RNA therapeutics, particularly siRNAs and antisense oligonucleotides (ASOs), depend on Watson-Crick base pairing for specificity. However, partial complementarity can lead to unintended interactions with non-target transcripts, resulting in off-target gene silencing or aberrant splice modulation (Jackson et al., 2016; Birmingham et al., 2006). Such off-target effects can cause cytotoxicity, immune activation, and phenotypic abnormalities, complicating clinical translation. Advancements in siRNA design algorithms, incorporating seed region analysis and thermodynamic profiling, have improved specificity (Reynolds et al., 2004; Jackson and Linsley, 2010). Transcriptome-wide off-target assessment via high-throughput sequencing and bioinformatics pipelines allows empirical identification and minimization of unintended interactions (Fedorov et al., 2006; Grimm et al., 2006).

Emerging RNA editing technologies, such as CRISPR-Cas13-based platforms, promise higher precision by targeting RNA transcripts without permanent genomic alterations (Abudayyeh et al., 2017; Cox et al., 2017). Nonetheless, comprehensive off-target profiling remains imperative to ensure safety (Wang et al., 2018). Although bioinformatic algorithms have improved siRNA and ASO specificity, transcriptome-wide analyses continue to reveal low-frequency but biologically significant off-target interactions (Jackson and Linsley, 2010; Fedorov et al., 2006). A critical challenge is that even rare events may translate into long-term safety risks in chronic settings. This highlights the need for standardized preclinical off-target screening pipelines and regulatory requirements for transcriptome-level safety profiling, which remain underdeveloped.

### 5.3 Manufacturing scale-up: process robustness, quality control, and global capacity

Manufacturing RNA therapeutics at scale introduces multifaceted challenges. The core RNA synthesis step—often *in vitro* transcription—must maintain sequence fidelity, high purity, and controlled capping and tailing to ensure functionality and safety (Kauffman et al., 2016; Pardi et al., 2018). Impurities such as double-stranded RNA contaminants can provoke unwanted immune activation, mandating rigorous purification protocols (Karikó et al., 2005a).

Formulation with delivery vehicles, primarily lipid nanoparticles (LNPs), requires reproducible encapsulation efficiency, particle size distribution, and stability to preserve therapeutic activity and facilitate regulatory approval (Kulkarni et al., 2018; Sago et al., 2018). Microfluidic manufacturing technologies have enabled scalable, controlled LNP production with consistent quality (Valencia et al., 2012).Global manufacturing capacity constraints were exposed during the COVID-19 pandemic’s mRNA vaccine rollout, underscoring the need for diversified and decentralized production infrastructure (Dolgin, 2021). Further innovation in continuous manufacturing, process analytical technologies (PAT), and supply chain robustness are essential to meet rising demand (Sharma et al., 2021).

Manufacturing challenges extend beyond cost and capacity. While LNP-based production has successfully scaled for vaccines, viral vector manufacturing remains less adaptable, constrained by packaging limits and complex purification processes (Naldini, 2015; Ginn et al., 2018). These differences underscore the importance of aligning platform choice with scalability requirements, particularly for global distribution. Without more robust and harmonized production pipelines, supply bottlenecks will continue to hinder equitable access.

### 5.4 Delivery challenges to extrahepatic sites

Several biological and physiological barriers impede RNA therapeutic delivery to extrahepatic organs. The vascular endothelium, tissue extracellular matrix, cellular membranes, and endosomal compartments each impose restrictions on RNA biodistribution and intracellular bioavailability (Sahay et al., 2010). The liver’s unique fenestrated endothelium facilitates nanoparticle and conjugate access to hepatocytes, a privilege not shared by many other tissues.

For CNS delivery, the blood-brain barrier (BBB) presents a formidable obstacle, restricting passage of most macromolecules including RNA-loaded nanoparticles. Strategies under investigation to circumvent the BBB include receptor-mediated transcytosis via transferrin or insulin receptors, intrathecal or intracerebroventricular administration, and design of ultrasmall or surface-modified nanoparticles capable of BBB penetration (Bicker et al., 2014; Saraiva et al., 2016). Nonetheless, clinical translation of CNS RNA therapeutics remains nascent, with Nusinersen—an intrathecally delivered ASO for spinal muscular atrophy—being a notable exception (Mercuri et al., 2018).

Targeting solid tumors and pulmonary tissues faces additional complexity due to heterogenous tumor vasculature, high interstitial pressure, and immune-suppressive microenvironments (Shi et al., 2020). Nanoparticle surface modifications with ligands targeting tumor-associated antigens or components of the tumor microenvironment offer promising approaches to enhance specificity and uptake (Bertrand et al., 2014). Pulmonary delivery of RNA via inhalable formulations is also being explored for respiratory diseases, but challenges in aerosol stability and mucociliary clearance remain (Lam et al., 2012).

Importantly, progress in extrahepatic delivery has been incremental rather than transformative. Most approved therapies remain liver-targeted, while CNS and solid tumor delivery are still experimental (Saraiva et al., 2016; Shi et al., 2020). This persistent gap suggests that delivery innovations should be evaluated not only for proof-of-concept efficacy but also for translational scalability, manufacturability, and safety in chronic settings—dimensions often overlooked in preclinical studies.

### 5.5 Long-term safety considerations

As RNA therapeutics transition from acute treatments to chronic disease management, long-term safety data become paramount. Chronic administration increases the risk of cumulative toxicity, immune sensitization, and unforeseen off-target effects (Setten et al., 2019). Immunogenicity, though mitigated by nucleoside modifications and delivery optimization, can evolve over prolonged exposure. Development of anti-drug antibodies or adaptive immune responses to delivery vehicle components may reduce efficacy or precipitate adverse reactions (Irvine et al., 2020). Potential genotoxicity and unintended genomic alterations are concerns particularly relevant to RNA-guided genome and epigenome editing platforms such as CRISPR-Cas9 and base editors. Although RNA editing is transient and does not alter DNA directly, delivery methods and off-target activity necessitate rigorous preclinical and clinical evaluation (Gillmore et al., 2021; Cox et al., 2017). Post-marketing surveillance programs, patient registries, and real-world data collection are critical to monitor long-term outcomes and rare adverse events, complementing pre-approval safety studies. Regulatory agencies emphasize the importance of such pharmacovigilance frameworks to ensure sustained patient safety.

## 6 Future directions and opportunities

**FIGURE 3:**
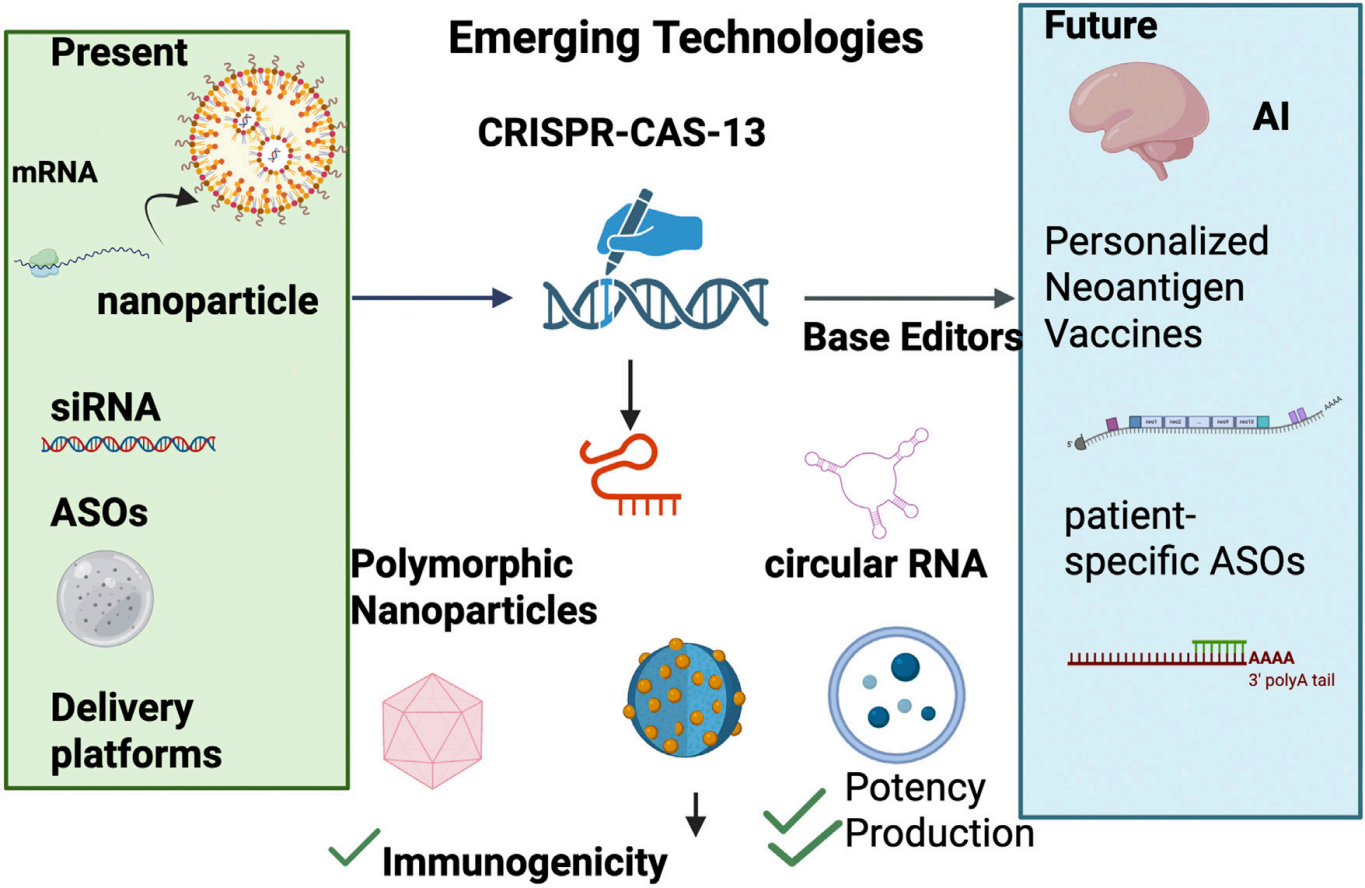
Roadmap of RNA therapeutics from present to future. The figure illustrates the progression of RNA therapeutics from established modalities to emerging technologies and future directions. On the left, current approaches include mRNA vaccines delivered by lipid nanoparticles, small interfering RNAs (siRNAs), and antisense oligonucleotides (ASOs), supported by established delivery platforms. In the center, emerging technologies are highlighted, such as CRISPR-Cas13 for programmable RNA editing, circular RNAs with enhanced stability, self-amplifying RNAs (saRNAs), and advanced nanoparticle formulations designed to improve immunogenicity, potency, and production. On the right, forward-looking innovations include artificial intelligence (AI)-guided sequence design, personalized neoantigen vaccines, and patient-specific ASOs, underscoring the potential for precision medicine and adaptive therapeutic strategies.

The landscape of RNA therapeutics is rapidly evolving, driven by transformative advances that promise to overcome current limitations and unlock new paradigms in precision medicine Figure 3. Future developments are centered around highly personalized treatment modalities, revolutionary RNA editing technologies, and the synergistic integration of artificial intelligence (AI) to optimize therapeutic design and clinical outcomes. This convergence is set to redefine the scope, efficacy, and accessibility of RNA-based interventions.

### 6.1 Personalized RNA therapies: tailoring to the individual molecular landscape

Personalization is emerging as the cornerstone of next-generation RNA therapeutics. Unlike traditional small molecules or protein biologics, RNA therapies offer unparalleled modularity—enabling rapid sequence customization to match individual genetic mutations, neoantigens, or disease-specific transcriptomic profiles (Sahin and Türeci, 2018). This capability is particularly crucial in oncology, where tumor heterogeneity and patient-specific mutational burdens necessitate bespoke immunotherapies.

Personalized mRNA cancer vaccines, which encode patient-specific neoantigens identified through tumor exome sequencing, exemplify this approach. Clinical trials have demonstrated that these vaccines elicit robust and durable cytotoxic T-cell responses, often synergizing with immune checkpoint inhibitors to overcome tumor immune evasion (Ott et al., 2017; Keskin et al., 2019). The ability to rapidly synthesize and clinically deploy patient-tailored mRNA constructs is facilitated by platform manufacturing, which standardizes non-sequence elements while allowing individualized payload variation (Pardi et al., 2018).

Beyond oncology, genetic diseases with rare or private mutations benefit from personalized RNA therapies. The case of *Milasen*—a splice-switching antisense oligonucleotide (ASO) developed for a single patient with Batten disease—demonstrates the feasibility of ultra-rapid, patient-specific RNA therapeutic design, manufacturing, and compassionate clinical use (Kim et al., 2019). This “n-of-1” approach challenges traditional drug development paradigms, raising regulatory, ethical, and manufacturing challenges that necessitate novel frameworks for evaluation and approval (Miller et al., 2020). Realizing widespread personalized RNA therapeutics will depend on scalable, agile manufacturing technologies, streamlined regulatory pathways that accommodate rapid design cycles, and advanced biomarker-guided patient stratification (Sahin et al., 2017).

### 6.2 Novel RNA editing technologies: precision beyond genetic code alteration

RNA editing technologies are revolutionizing the therapeutic landscape by enabling transient, reversible, and highly specific modifications of RNA transcripts without altering the underlying genomic DNA, thus offering safety advantages over permanent genome editing (Cox et al., 2017).

CRISPR-Cas13 systems, distinct from the DNA-targeting Cas9, provide programmable RNA cleavage and modulation capabilities. Cas13 effectors can be fused to RNA editing enzymes to facilitate base conversions (e.g., adenosine-to-inosine) that correct point mutations at the transcriptome level, thereby restoring protein function (Abudayyeh et al., 2017; Konermann et al., 2018). These RNA-targeting systems minimize risks of off-target DNA damage and heritable changes, offering a therapeutic advantage for transient or dosage-dependent disease modulation (Liu et al., 2017).

Moreover, base editors such as REPAIR and RESCUE enable single-nucleotide modifications with high specificity, broadening the therapeutic window for diseases caused by point mutations (Zhang et al., 2017). Delivery of these complex editing constructs remains a critical bottleneck; lipid nanoparticle encapsulation and adeno-associated viral vectors are being optimized for tissue-specific delivery and immune evasion (Gillmore et al., 2021). Ethical and safety considerations surrounding RNA editing include immunogenicity of CRISPR components, off-target RNA binding, and unintended splice variants. Ongoing preclinical and clinical studies aim to characterize and mitigate these risks (Liang et al., 2017). Beyond editing systems, additional RNA modalities such as circRNAs, lncRNAs, riboswitches, and RNA-targeting small molecules are expanding the therapeutic landscape; each bringing distinct opportunities and challenges for translation.

Taken together, these challenges are unevenly distributed across RNA modalities. Immunogenicity is a well-characterized concern for mRNA and saRNA vaccines, while CRISPR-based editors remain in earlier stages of evaluation where long-term safety and immune responses are less defined. Manufacturing scalability is largely solved for mRNA/LNPs, remains cost-intensive for AAV, and is still immature for exosome-based systems. Delivery beyond the liver continues to limit all platforms, with GalNAc confined to hepatocytes and polymers or exosomes showing only exploratory promise. Long-term safety data are strongest for ASOs and siRNAs, whereas newer platforms such as RNA editors and circRNAs lack longitudinal follow-up. These cross-modal comparisons are consolidated in Table 3, which links each challenge to the modalities most affected and the maturity of the supporting evidence.

#### 6.2.1 Under-explored RNA modalities: mechanisms and translational outlook

CircRNAs and saRNAs enhance durability and dose-sparing but require advances in design and manufacturing (Blount and Breaker 2006). LncRNA-targeting approaches and RNA-focused small molecules broaden the target space but hinge on delivery precision and specificity. Riboswitches provide the prospect of built-in control mechanisms for therapeutic RNAs, contingent on achieving robust ligand–switch pairs (Wilson et al., 2023; Finkel et al., 2017; Frangoul et al., 2021; Jackson et al., 2006; Zhao et al., 2020).

##### 6.2.1.1 Circular RNAs (circRNAs)

CircRNAs are covalently closed RNA molecules that resist exonuclease degradation and can support prolonged translation when engineered with internal ribosome entry sites or synthetic initiation elements. Compared with linear mRNA, circRNAs may enable more durable protein expression and reduced innate immune activation, making them attractive for oncology vaccines and enzyme replacement strategies. However, large-scale production remains a challenge due to sequence design requirements and the need to eliminate linear RNA by-products during purification (Wesselhoeft et al., 2018; Orna Therapeutics, 2024).

##### 6.2.1.2 Long non-coding RNAs (lncRNAs)

LncRNAs regulate gene expression through scaffolding, chromatin remodeling, and by acting as molecular decoys. Their therapeutic applications are primarily as targets for knockdown via antisense oligonucleotides (ASOs) or siRNAs, with potential in oncology, cardiovascular, and metabolic disease. The main translational barriers are incomplete functional annotation, context-dependent expression, and the challenge of efficient, cell-type-specific delivery (Statello et al., 2021).

##### 6.2.1.3 Riboswitches

Riboswitches are cis-acting RNA elements that alter their conformation to regulate splicing or translation upon ligand binding. When engineered into therapeutic constructs, riboswitches could provide drug-responsive, tunable control of RNA activity, potentially reducing systemic exposure. Translation to the clinic, however, depends on discovering high-affinity and selective small-molecule ligands, and on demonstrating that switch integration does not impair RNA stability or expression (Breaker, 2022).

##### 6.2.1.4 RNA-targeting small molecules

Beyond oligonucleotide-based drugs, small molecules can modulate RNA structure, splicing, or translation. Several orally bioavailable RNA-targeting compounds are in development, including splicing modifiers for neuromuscular disorders. The challenges are selectivity among structurally similar RNA motifs and the risk of widespread off-target effects. Advances in structure-guided design and transcriptome-wide profiling will be critical to progress in this area (Meyer et al., 2020).

##### 6.2.1.5 Net assessment

Together, these modalities extend the RNA therapeutic landscape in complementary ways. CircRNAs and saRNAs enhance durability and dose-sparing but require advances in design and manufacturing. LncRNA-targeting approaches and RNA-focused small molecules broaden the target space but hinge on delivery precision and specificity. Riboswitches provide the prospect of built-in control mechanisms for therapeutic RNAs, contingent on achieving robust ligand–switch pairs. Their eventual success will depend on both technological refinement and integration with delivery systems adaptable to diverse RNA structures. Realizing the full potential of these emerging modalities will require not only advances in chemistry and delivery, but also the integration of computational approaches capable of guiding design, predicting outcomes, and tailoring therapies to individual patients—an area where artificial intelligence is beginning to play a transformative role.

### 6.3 Integration with AI-Driven precision medicine: accelerating design and clinical decision-making

Artificial intelligence (AI) and machine learning (ML) technologies are fundamentally transforming RNA therapeutic development by enabling data-driven design, predictive modeling, and personalized treatment strategies. AI algorithms facilitate rational design of RNA sequences that maximize target affinity while minimizing immunogenicity and off-target effects, leveraging deep learning models trained on extensive datasets of RNA structure, function, and biological interactions. These computational tools reduce iterative laboratory experimentation, accelerating candidate optimization and reducing development timelines.

Recent studies highlight the tangible benefits of AI integration in RNA therapeutic development. For example, deep learning–based siRNA design platforms have reduced predicted off-target binding by up to 40%–60% compared with traditional rule-based algorithms. Similarly, generative AI models have been applied to optimize mRNA secondary structure, resulting in twofold improvements in protein translation efficiency in preclinical assays. Machine learning frameworks have also predicted LNP biodistribution patterns with >80% accuracy, accelerating formulation development and reducing empirical trial-and-error. These case studies illustrate that AI not only accelerates discovery but also quantitatively improves therapeutic design and delivery performance.

In delivery system development, ML models predict nanoparticle physicochemical properties, biodistribution patterns, and cellular uptake efficiency, enabling *in silico* screening of formulation parameters. This reduces reliance on costly and time-intensive empirical trials. Clinically, AI supports patient stratification through integration of multi-omics data, electronic health records, and imaging, identifying responders to RNA therapeutics and predicting adverse events (Topol, 2019). Adaptive trial designs powered by AI enhance statistical power and personalized dosing regimens, improving therapeutic outcomes and reducing trial failures. The future convergence of RNA therapeutics with AI-driven precision medicine heralds an era where RNA drugs are designed, delivered, and dosed with unparalleled specificity, heralding a new paradigm of individualized healthcare.

## 7 Conclusion

The journey of RNA therapeutics, from foundational discoveries in molecular biology to transformative clinical applications, exemplifies the power of innovation in modern medicine. What began as pioneering work on antisense oligonucleotides and RNA interference has blossomed into a versatile platform capable of modulating gene expression with remarkable precision and flexibility (Zamecnik and Stephenson, 1978; Fire et al., 1998). The extraordinary success of mRNA vaccines during the COVID-19 pandemic has served not only as a landmark achievement but as a catalyst accelerating research, development, and public trust in RNA-based technologies (Baden et al., 2021; Pardi et al., 2018).

At the heart of RNA therapeutics lies its unique adaptability. Unlike traditional therapies, RNA drugs can be rapidly designed, synthesized, and tailored to individual molecular signatures, paving the way for personalized medicine that addresses the heterogeneity of diseases such as cancer and rare genetic disorders (Sahin and Türeci, 2018; Kim et al., 2019). Personalized RNA vaccines targeting patient-specific neoantigens have demonstrated encouraging clinical efficacy, illustrating a new paradigm where immune responses can be precisely shaped to individual tumor profiles (Ott et al., 2017; Keskin et al., 2019).

Technological breakthroughs in delivery vehicles, including lipid nanoparticles and targeted conjugates, have resolved longstanding barriers of stability, immunogenicity, and tissue specificity, enabling safe and effective *in vivo* administration of RNA molecules (Cullis and Hope, 2017; Kulkarni et al., 2018). Yet, challenges remain—delivery to extrahepatic tissues such as the central nervous system, lungs, and solid tumors demands further innovation, and the complexities of immune activation and off-target effects require ongoing refinement (Gillmore et al., 2021).

Emerging RNA editing technologies offer a new layer of therapeutic finesse, enabling transient, reversible, and highly specific transcript modifications that sidestep permanent genomic alterations and their associated risks (Cox et al., 2017; Pickar-Oliver and Gersbach, 2019). These platforms, combined with the accelerating influence of artificial intelligence and machine learning, are reshaping the landscape of RNA drug design and clinical decision-making. AI-driven models enhance target identification, optimize sequence and delivery design, and facilitate personalized treatment regimens, heralding an era of data-informed precision therapeutics (Topol, 2019).

In conclusion, the field of RNA therapeutics is moving rapidly from early proof-of-concept studies toward addressing the practical challenges that will determine its long-term success in the clinic, a trajectory that is consistent with recent analyses highlighting advances in mRNA nanomedicine, molecular stability, and delivery systems as pivotal for broadening therapeutic applications (Dwivedi et al., 2024; Dwivedi et al., 2025). Such insights reinforce the importance of focusing future efforts not only on conceptual innovation but also on the translational bottlenecks that currently limit clinical scalability. The next decade must prioritize four critical areas. First, the establishment of scalable and globally distributed manufacturing platforms capable of rapidly pivoting to personalized therapies or pandemic-responsive applications will be essential for timely and equitable access (Dolgin, 2021; Sharma et al., 2021). Second, innovations in delivery systems for extrahepatic tissues—including the central nervous system, lungs, and solid tumors—are needed, leveraging engineered nanoparticles, exosomes, and ligand-based conjugates to expand therapeutic reach (Bicker et al., 2014; Saraiva et al., 2016; Bertrand et al., 2014). Third, the development of long-term safety frameworks will be crucial, particularly for repeat dosing regimens and for emerging RNA-editing platforms where off-target risks and immune responses remain incompletely characterized (Setten et al., 2019; Gillmore et al., 2021). Finally, the integration of artificial intelligence into RNA drug development offers powerful opportunities to optimize sequence design, delivery formulations, and adaptive clinical trial strategies. By evaluating RNA classes and delivery systems against these dimensions, a more nuanced picture emerges. Small interfering RNAs and GalNAc conjugates have reached a level of clinical maturity and reproducibility but remain confined to hepatocyte targets (Adams et al., 2018; Ray et al., 2020). Messenger RNAs, validated during the COVID-19 pandemic, demonstrate remarkable scalability yet require innovation in tissue targeting and immune modulation (Polack et al., 2020; Baden et al., 2021). Circular RNAs and self-amplifying RNAs offer unique opportunities for sustained or dose-sparing expression, though their production and quality control remain under development (Wesselhoeft et al., 2018; Bloom et al., 2021). CRISPR-based RNA editing and targeting approaches hold great promise for precision therapies, but their translation depends on solving outstanding questions around delivery fidelity and long-term genomic stability (Gillmore et al., 2021). Beyond these, underexplored modalities—including long noncoding RNAs, riboswitches, and RNA-targeting small molecules—are beginning to reveal therapeutic potential and deserve greater integration into future strategies (Statello et al., 2021; Meyer et al., 2020).

Taken together, this synthesis underscores where evidence already supports clinical translation and where critical gaps remain, while offering a forward-looking perspective that identifies realistic priorities for advancing RNA-based interventions from experimental promise to widely accessible precision medicines.
